# Research- and Practice-Based Nutrition Education and Cooking Workshops in Pediatric Oncology: Protocol for Implementation and Development of Curriculum

**DOI:** 10.2196/resprot.8302

**Published:** 2018-01-09

**Authors:** Cynthia Chaput, Sabrina Beaulieu-Gagnon, Véronique Bélanger, Simon Drouin, Laurence Bertout, Lucie Lafrance, Cinthia Olivier, Marthe Robitaille, Caroline Laverdière, Daniel Sinnett, Marie Marquis, Valérie Marcil

**Affiliations:** ^1^ Department of Nutrition Faculty of Medicine Université de Montréal Montreal, QC Canada; ^2^ Research Center of Sainte-Justine University Health Center Montreal, QC Canada; ^3^ Institute of Nutrition and Functional Foods Laval University Quebec City, QC Canada; ^4^ Division of Hematology-Oncology Sainte-Justine University Health Center Montreal, QC Canada; ^5^ Department of Pediatrics Faculty of Medicine Université de Montréal Montreal, QC Canada

**Keywords:** child, diet, education, neoplasms, hospitals, methods

## Abstract

**Background:**

Progresses in childhood cancer treatment, diagnosis, and management have resulted in childhood cancer survival rates of over 80%. However, this therapeutic success comes with a heavy price: two-thirds of childhood cancer survivors will be affected by further complications, including cardiovascular and metabolic diseases. Adequate nutrition during cancer treatment is essential to ensure the child’s optimal development, improve tolerance to treatments, and can contribute to lower the risk of developing cardiometabolic diseases. Side effects of cancer treatments can negatively impact children’s nutritional intake and eating behaviors. Involving the families of childhood cancer patients in educational workshops could be a promising avenue to promote healthy eating during and after cancer treatment.

**Objective:**

The objectives of this study were to develop, validate, and implement a family-based nutrition education and cooking workshop curriculum in a pediatric oncology setting that addresses the nutritional issues encountered during treatments while promoting the adoption of healthy eating habits for the prevention of long-term cardiometabolic effects.

**Methods:**

The workshops were developed and validated following an 8-step iterative process, including a review of the literature and consultations with a steering committee. An evaluation tool was also developed. A nonrandomized study protocol was elaborated to implement the workshops and measure their impact. The themes of the 6 research- and practice-based lessons are as follows: meal fortification during cancer treatment, changes in taste during cancer therapy and their impact on children, adapting diet to eating-related side effects of treatments, nutritional support during cancer treatment, Mediterranean diet and health, and planning quick and economic meals. The validation process included consultations with the institution’s clinical nutrition professionals. Self-administered post questionnaires were developed according to the content of each workshop to measure short-term outcomes, namely, participants’ perception of knowledge acquisition, behavioral intention, and satisfaction. Medium-term outcomes that will be evaluated are participants’ anthropometric profile, quality of the diet, and circulating biomarkers of metabolic health.

**Results:**

The project was funded in 2016 and enrollment will be completed in 2021. Data analysis is currently under way and the first results are expected to be submitted for publication in 2019.

**Conclusions:**

This research- and practice-based nutrition education and cooking demonstration curriculum could be a valuable complement to a multidisciplinary lifestyle intervention for the prevention of long-term cardiometabolic complications in childhood cancer.

## Introduction

### Long-Term Health Complications in Childhood Cancer Survivors

Progresses accomplished in childhood cancer treatment, diagnosis, and management in the past decades have led to survival rates exceeding 80% [1,2]. Despite these encouraging statistics, it is estimated that about two-thirds of childhood cancer survivors (CCS) will be affected by treatment-related long-term complications [3], including cardiovascular and metabolic diseases [4,5]. Lifestyle practices such as healthy eating and physical activity are well-recognized modifiable factors that contribute to lower the risk of cardiometabolic complications [6]. Particularly, adherence to a Mediterranean dietary pattern has been associated with reduced risk factors related to the metabolic syndrome in survivors of childhood acute lymphoblastic leukemia [7]. In addition to the prevention of long-term sequelae, good nutrition is essential to ensure children’s requirements for growth and development during cancer treatments. Adequate nutritional status is also associated with increased tolerance to cancer treatments, better prognosis, and enhanced quality of life [8]. Side effects of cancer treatments such as nausea, mucositis, taste disorders, poor appetite, or increased appetite secondary to steroids intake can impact children’s eating behaviors and nutritional status [8]. Furthermore, it is known that eating habits acquired in childhood are likely to persist in adulthood [9,10] and after completion of cancer treatments [11].

### Children’s Dietary Habits During Cancer Treatment

Studies on CCS have described similar dietary habits to those of the general population, reflecting a suboptimal diet for the prevention of metabolic syndrome components such as obesity, insulin resistance, arterial hypertension, or dyslipidemia [12]. So, involving families of children with cancer to adopt or maintain healthy habits during and after cancer treatment is essential. Moreover, given that parents often experience time constraints [13] and economic burden related to transportation, accommodation, or loss of work income [14], practical advice for meal preparation should be provided to meet families’ needs. Design and evaluation of family-based nutrition and cooking education programs are increasingly reported in the literature, mainly related to the prevention or management of childhood obesity [15-19]. Nutritional interventions for young CCS and their families have been described in the literature [20-22], but, to our knowledge, only few were developed for children undergoing cancer treatment and those that were targeted patients in the maintenance phase of therapy [23-25]. As there is a need to develop and evaluate the feasibility of an early intervention during the course of pediatric cancer treatments, we have developed 6 nutrition and cooking education workshops that aim to increase knowledge relative to the following: (1) children’s nutrition during and after cancer treatments; (2) healthy, quick, and economical food preparation, cooking techniques, and food safety specific for children with cancer; and (3) development of food preferences during childhood and parental feeding practices. Here, we describe the following: (1) the development and validation of the workshops; (2) the elaboration of an evaluation tool; and (3) the study protocol to implement the workshops and to measure their impact.

## Methods

### Setting

We have developed a protocol for a nonrandomized controlled study. This study has been developed within the VIE (Valorization, Implication, Education) Program at the Sainte-Justine University Health Center (SJUHC) in Montreal, Canada. This research program consists of a 4-year family-oriented multidisciplinary interventional program integrating physical activity, nutrition, and psychosocial clinical and research teams. The nutritional intervention includes personalized assessment, goal setting, and counseling for behavioral changes with a registered dietitian (RD) as well as group nutrition education and cooking workshops providing complementary information. The SJUHC Institutional Review Board approved the study, and investigations will be carried out in accordance with the principles of the Declaration of Helsinki.

### Curriculum Development and Validation Process

The research- and practice-based curriculum consists of 6 lessons developed by a team of researchers and RD and validated by the hematology-oncology clinical nutrition team at SJUHC. The workshops are designed to provide reliable, up-to-date nutritional information geared to address specific themes and associated to cooking demonstrations facilitated by a RD and a chef.

To develop the curriculum, evidence for common nutritional and behavioral eating problems related to side effects of childhood cancer treatment and their management has been reviewed in the scientific literature published between 2000 and 2017 contained in Medline, PubMed, and Scopus databases. A few core papers published before 2000 have also been considered. Gray literature was searched for Canadian governmental guidelines and family-oriented documentation related to children’s diet while on cancer treatment published by recognized organizations, such as the Children Oncology Group and the Canadian Cancer Society. Insight from the SJUHC Centre de cancérologie Charles-Bruneau (CCCB) clinical nutrition team has also been sought. Recipes for demonstration were developed and standardized to match each of the 6 lesson themes. Nutritional value of recipes was analyzed based on general (for protein, lipid, and sodium content) and theme-specific criteria and was inspired by those of the SJUHC institutional food service, of early childhood nutrition reference [26], and of the Heart and Stroke Foundation program [27].

**Figure 1:**
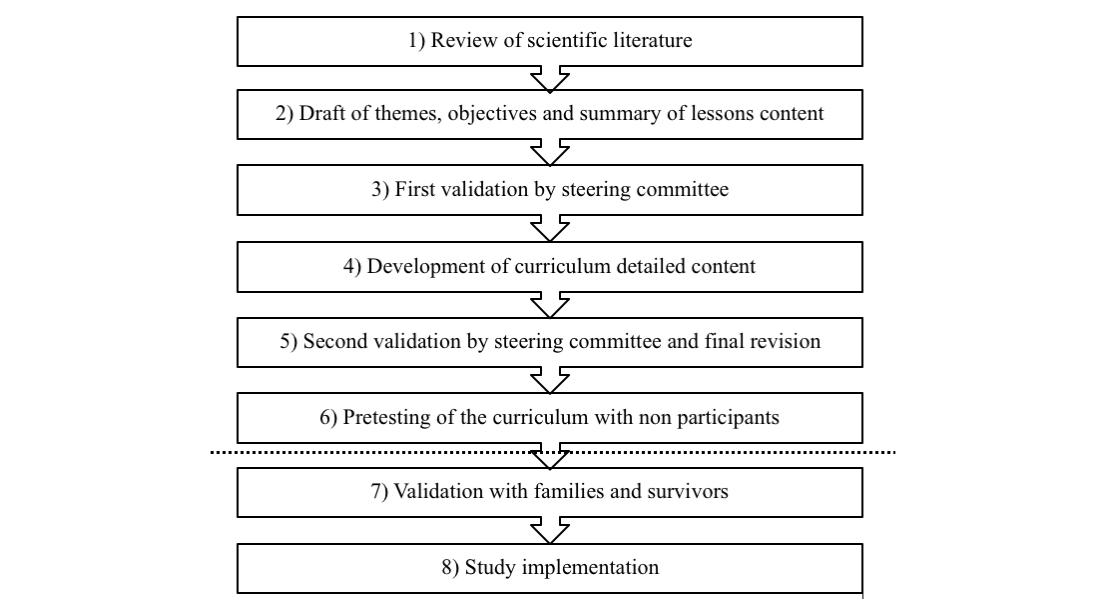
The 8-step development and validation process of the VIE (Valorization, Implication, Education) Program educational workshops. The dotted line divides the steps that have been completed and those to be performed.

The curriculum was validated concurrently with its development within an 8-step process (Figure 1). The first 6 steps have already been completed. Subsequent to the literature review, primary themes and specific lesson objectives were elaborated and submitted to a steering committee, composed of SJUHC CCCB clinical RD (n=2) and the Department head of the Clinical Nutrition Services, representing, respectively, 19, 15, and 10 years of experience in pediatric oncology. A consensus on improvements to be made was obtained, and a detailed content of the lessons was then elaborated based on current scientific evidence and common practices by the clinical nutrition team. The modified and detailed content was submitted to the clinicians for a second validation, followed by a final revision of the curriculum. Workshops were pretested with nonparticipants, including CCCB health care professionals, leading to further refinements.

Subsequent steps will be performed before official implementation and intervention evaluation. A validation meeting will be conducted with a committee composed of family members and CCS to better tailor the curriculum regarding the length of the workshop, the timing, and the amount of information delivered. After implementation, we will continually monitor the curriculum and evaluation tools to fine-tune and optimize content delivery.

### Curriculum Description and Content

The developed curriculum consists of 6 themed workshops with specific objectives and key messages (Table 1). The 6 lessons are independent from one another, and there is no specific sequence to attend the workshops. This will increase convenience and allow flexibility according to participants’ schedule and needs for information. The nutritional criteria of the recipes are presented in Table 2.

The curriculum will promote a liberalized diet that reinforces food safety guidelines for immunocompromised patients based on Health Canada food safety guidelines for people with a weakened immune system [61] and in line with the SJUHC CCCB standard clinical practices. Patients receiving hematopoietic cell transplantation will be referred to the hematology-oncology RD for personalized diet and food safety guidelines.

**Table 1:** Themes, objectives, and evidence-based key messages of the nutrition education and cooking workshops.

Lesson theme	Specific objectives	Learning objectives (specific key messages)
Meal fortification during cancer treatment	Understand the role and importance of proteins, calcium, and vitamin D Learn strategies to fortify usual foods with these nutrients	Proteins are essential for tissue growth and repair and to support immune system function [28,29] A source of protein should be included in every meal [30] Calcium, vitamin D, and proteins are essential for bone growth and play a role in secondary osteoporosis prevention [31,32]
Changes in taste during cancer therapy and their impact on children	Understand the development of taste and food preferences in children Learn strategies to enhance the flavor of food and to improve meal acceptability during cancer treatment Understand positive parental feeding practices for optimal taste development and eating behaviors	Food aversion, neophobia, need for routine and security, need for self-expression, and learning from social modeling are normal behaviors related to the development of taste in children [9,33]. These behaviors can be affected by cancer treatments [34,35] Parental strategies and attitudes can influence adherence to a healthy diet in children during and after treatments. These include promoting participation in meal preparation when possible [36], maintaining a pleasant atmosphere during meal times, proposing a variety of food, and offering a mealtime structure (where, when, what), while letting the child decide the amount and select the foods from the offering [33] Some herbs, spices, and acidic food can enhance the flavor of dishes and can be used to mask the perceived metallic taste [37,38]
Adapting diet to eating-related side effects of treatments	Learn how to adapt the child’s diet to improve food intake when mucositis, nausea, or vomiting are present Learn strategies to attenuate diarrhea and constipation secondary to cancer treatments	Nausea during treatments can be a side effect of the treatment and can be caused by a metallic or a medication taste in the mouth (dysgeusia) [39,40] Strategies to adapt diet and promote oral intake when the child is experiencing mucositis include serving warm meals and nonirritating foods with soft and moist texture [41] Soluble fibers, notably psyllium, can be helpful for diarrhea, whereas insoluble fibers and concentrated sugars should be limited [42,43] Total dietary fibers promote intestinal regularity and help prevent constipation [43]
Nutritional support during cancer treatment	Demystify oral, enteral, and parenteral nutritional support to facilitate their acceptability by patients and families Understand positive parental feeding practices during nutritional support	Nutritional support is an adjuvant to cancer treatment in situations when the child’s needs are not met with oral eating alone [44,45] When allowed by the medical team, presentation of food to the child should be encouraged during nutritional support [46,47] Some strategies can facilitate acceptation of nutritional support [36,48]
Mediterranean diet and health	Learn approaches to integrate principles of the Mediterranean diet into usual meals Learn the benefits of adherence to a Mediterranean diet for the whole family	The Mediterranean diet brings health benefits to the whole family, especially for the prevention of cardiovascular diseases [49,50] The adherence to a Mediterranean diet can be improved with small changes daily (eg, adding a portion of vegetables to usual meals, replacing refined grains by whole grains) [7] Vegetal and animal proteins offer different health advantages: it is beneficial to diversify protein sources [51,52] The use of vegetable oils (nonhydrogenated) is preferred to butter or shortening [53,54]
Planning quick and economic meals	Learn planning strategies to remove barriers to cooking at home Learn tactics to prepare simple and quick meals using accessible and nutritious ingredients Acquire strategies for eating healthy on a budget	Meal planning saves time and reduces daily stress [55,56] Keeping some essential foods in the pantry, fridge, and freezer helps to prepare last-minute balanced meals [57] Low-cost alternatives can be found in several food categories [58,59]
		
		

**Table 2:** Nutritional criteria for recipes of the nutrition education and cooking workshops.

Lesson theme	Nutritional criteria^a^
Meal fortification during cancer treatment	Recipes rich in proteins that include at least one calcium-rich and one vitamin D-rich ingredient Protein: >20 g for a meal and >10 g for a snack Calcium: >165 mg for a meal and >0 mg for a snack Vitamin D (if possible): >15% of the adequate intake: 90 UI (2.25 mg)
Changes in taste during cancer therapy and their impact on children	Recipes include ingredients to enhance the taste of dishes (eg, herbs or spices) while limiting sodium and dietary fat and include ingredients to mask metallic taste (eg, acidic ingredients like lemon juice or vinegar)
Adapting diet to eating-related side effects of treatments	Recipes for nausea include: Cold or warm meals that release less odor Ingredients to enhance taste and mask metallic taste, such as herbs, spices, or acidic ingredients Recipes for diarrhea include: Meals without irritants (eg, strong spices, insoluble fiber) Soluble fibers: >2 g Limited in concentrated sugar: <5 g for a meal and <2 g for a snack Recipes for constipation include: Fiber-rich ingredients (eg, whole grains, vegetables, fruits) Total fibers >4 g Recipe for mucositis include: Dish with a soft and moist texture Without irritants (eg, strong spices, acidic ingredients, salt) Can be reduced in puree if needed Served at room temperature
Nutritional support during cancer treatment	Recipes rich in proteins (>20 g for a meal and >10 g for a snack) Meal or snacks also include complex carbohydrates and healthy fats
Mediterranean diet and health	Fish as the main ingredient Recipes include whole grains and vegetables, or suggest them as side dishes Include healthy fats (eg, canola or olive oil, nuts or seeds, avocado)
Planning quick and economic meals	Recipe includes 2 pantry essentials and costs less than Can $4 per portion

^a^Nutritional criteria are based on adult portions. Parents will be advised to adapt the portion served according to the child’s usual appetite. According to the Satter Eating Competence Model, the parent decides the type of food served while letting the child decide the amount based on his or her internal cues [33,60].

### Study Protocol

#### Recruitment of Participants and Controls

From January 2018 to December 2020, parents and children newly diagnosed with cancer, treated at the SJUHC, and meeting the inclusion criteria will be offered to participate in the VIE Program. Participant recruitment will be sequential. Inclusion criteria are as follows: (1) being less than 21 years old at diagnosis, (2) being treated with radiotherapy or chemotherapy (including patients receiving hematopoietic cell transplantation), and (3) able to give an informed consent (by parents or legal tutors). Participants who are not receiving radiotherapy or chemotherapy will be excluded from the study. Patients will be followed for 4 years. On average, 140 children per year are admitted at the SJUHC’s CCCB, of which about 110 would be eligible. On the basis of earlier studies, we expect an average of 75 patients recruited per year (70.0% recruitment rate), for a total of 150 participants over 2 years. Enrolled participants and their family (parents, grandparents, etc) will be encouraged to attend the nutrition education and cooking workshops. It is to the parents’ discretion to attend workshops with or without the child, according to the child’s age, interest, and health condition. The control group will be recruited sequentially from patients diagnosed at the CCCB 3 to 4 years ago, who were not exposed to the VIE Program and who fulfill the same inclusion criteria as for the intervention group. No intervention will be offered to control participants. The measures and questions used will be the same as for the end-of-protocol intervention in the patients from the intervention group.

#### Delivery of Educational Workshops

At first, the workshops will be offered in French considering that a majority of patients treated at the SJUHC CCCB are French speaking, but they will eventually be translated and offered in English. The lessons will be dispensed on a weekly basis. Weekly rotation of the 6 themed lessons and variable scheduled day and time will contribute to maximize participants’ exposure and participation. A total of 40 workshops will be offered each year for 4 years. The workshops will take place at the SJUHC CCCB in a room designed for this purpose. Participants will be invited to taste the demonstrated recipes at the end of the workshop, and printed material will be distributed, including recipes and key messages. Because the mean age of patients treated at the CCCB is 7 years old, additional food-related activities are planned for young children. A signature sheet will be used to record attendance at each session. Videotaped workshops will also be made available to participants on a secure Web platform. Posters will advertise the schedule and topics of the workshops, and the clinical team will receive reminders of the upcoming workshops so they can promote attendance.

#### Workshop Evaluation Tools and Outcome Measurement

A total of 6 self-administered post-intervention questionnaires were developed to measure short-term outcomes of the workshops, namely, perception of knowledge acquisition, behavioral intention, and satisfaction. At the end of each workshop, adults and children of 12 years and older will be asked to fill out a printed version of the lesson-specific questionnaire available in both French and English. To reduce the burden of participants, the questionnaires contain limited number of items and can be answered in a few minutes. The questionnaires have been reviewed by an expert in the field of program evaluation and were pretested with the target population to validate their comprehension and literacy [62].

These questionnaires will measure participants’ perception of knowledge acquisition based on the corresponding workshop’s key messages [63]. This measure based on the perception was preferred to a scholastic questionnaire that measures knowledge to reduce participants’ burden. Overall, 3 to 4 items are presented in the form of a statement derived from the learning objectives (key messages) and begin with “I have learned.” Participants will answer according to their degree of agreement: “I agree,” “I agree more or less,” “I don’t agree,” or “I already knew this information.” Additional questions about the intention to try the recipes at home or to use the information to adapt the child’s diet [62] are also included. Participants’ relationship with the patient (patient, parent, grandparent, etc) consists in the only sociodemographic item captured by the questionnaire. General satisfaction will be measured by asking about the intention to recommend the workshop to others and, if not, to specify why. Finally, a comment section will enquire for qualitative feedback and for suggestions to improve the curriculum.

An activity report to be filled out by the facilitators after each session has also been developed to assess fidelity to the developed curriculum, to record attendance at each session, and to document any adverse event that could have disturbed the group or compromise content transmission. Qualitative feedback from facilitators related to challenges experienced during the workshops will also be sought in the activity report. These short-term outcome data will be used for evaluating the educational workshops.

Medium-term outcomes of the workshops as a component of the nutrition program will be measured as part of the evaluation of the broader controlled study of the VIE Program. Data will be collected by research staff at the beginning, during, and at the end of the intervention for each participant in the program. They will include socio-demographic data (ethnicity and socio-economic status), clinical data (age, diagnosis, age at diagnosis, and treatment protocol), anthropometric profile (body mass index, tricipital and subscapular skinfolds, head circumference [for children <3 years old]), and biomarkers of metabolic health (fasting lipid profile, glucose, insulin, and glycated hemoglobin). Diet will be assessed by an RD using 24-hour diet recalls and food journals. Diet data will be analyzed using the software Nutrific (Department of Food Science and Nutrition, Université Laval, Montreal, Canada). Nutrient values from this application are derived from the 2010 Canadian Nutrient File. Participation in workshops will be assessed using an attendance sheet at each workshop and by questioning participants and their family, at RD follow-up visits, the workshops attended or viewed on the Web platform, and their topics. Quality of diet and anthropometric and biochemical profiles will be analyzed in relation to workshop attendance and will be compared with those of the control group who did not participate in the workshops. Qualitative data regarding usefulness of each workshop will also be collected through focus groups of workshops’ participants. They will be leaded by the research RD and will take place at the end of selected sessions for all 6 thematic workshops. Collected data will be subject to thematic analysis to better understand if attendance to specific sessions is related to participants’ success.

**Figure 2:**
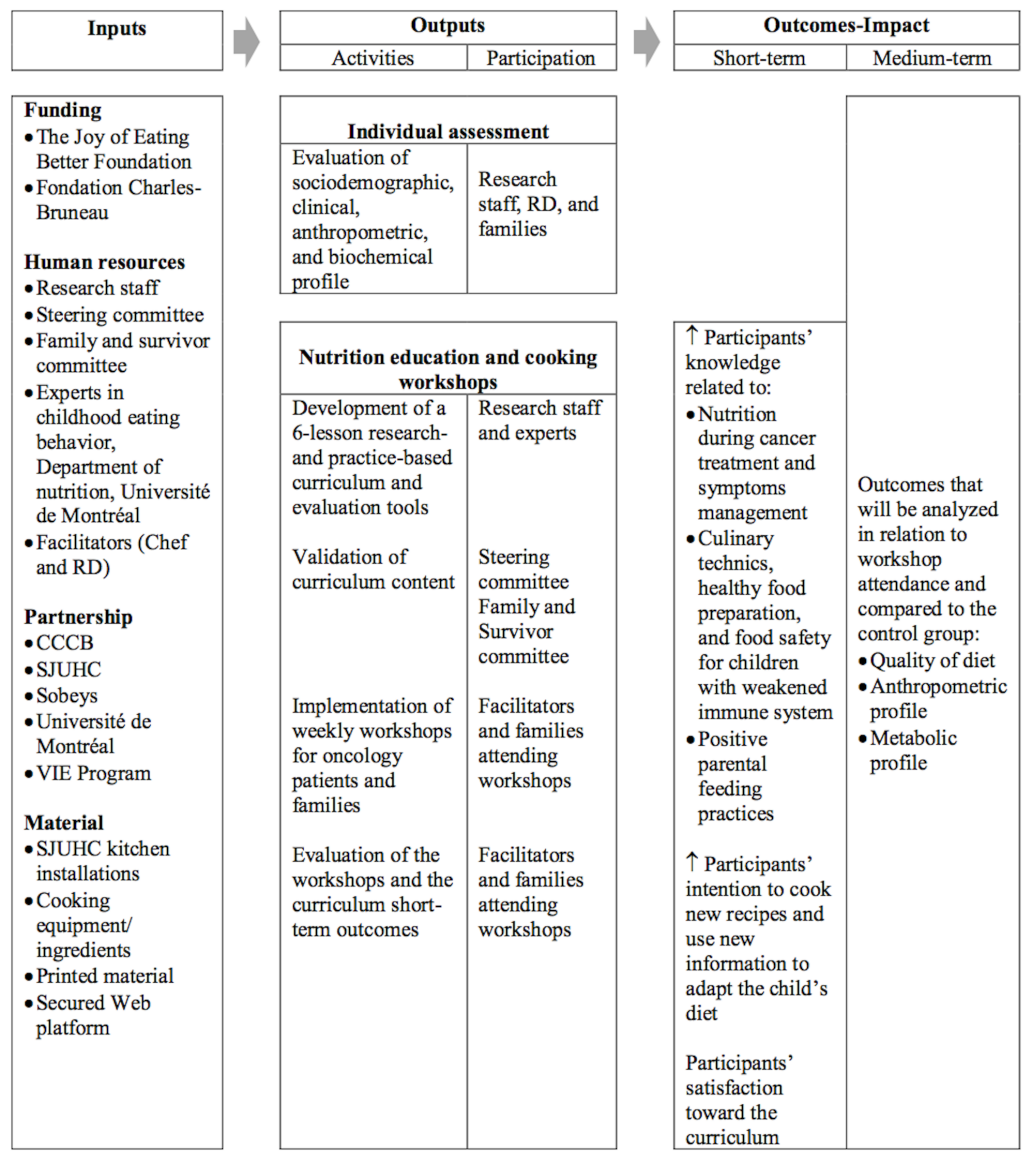
Logic model of the VIE (Valorization, Implication, Education) Program educational workshops. SJUHC: Sainte-Justine University Health Center; RD: registered dietitian; CCCB: Centre de cancérologie Charles-Bruneau.

A logic model [64,65] has been developed describing the resources needed (inputs), the activities achieved or to be implemented, the public reached (outputs), and the expected short- and medium-term outcomes of the VIE Program educational workshops (Figure 2).

## Results

The project was funded in 2016 and enrollment will be completed in 2021. Data analysis is currently under way and the first results are expected to be submitted for publication in 2019.

## Discussion

### Development of the Intervention

With this study, we have developed a family-oriented nutrition education and cooking workshop curriculum specific to pediatric oncology. The elaboration based on scientific evidence and on years of clinical experience, combined with an 8-step validation process, are strengths and features of interest of this study [64,66]. There is a consensus on the value of including field actors and representatives of the target population in the development of lifestyle interventions. Including clinicians in the development process offers precious insight to enhance the curriculum content and ensures coherence with the medical team. In CCS overweight children, a group lifestyle intervention used interviews and focus groups with health care providers and CCS parents to adapt a curriculum previously shown to be effective in non-CCS overweight children [67].

Some authors suggested that adding a cooking component to nutrition education is a good way to enhance participants’ skills and increase application of knowledge [68,69]. In our curriculum, cooking demonstrations will allow observational learning [68] and may enhance participants’ familiarity with specific foods [70], cooking techniques, and food safety practices. Moreover, the nutrition education content will focus on practical application as several ideas to apply recommendations and tips to overcome barriers to healthy eating and home cooking will be presented. Furthermore, the developed content aims at reinforcing the messages conveyed by the clinical and research RD during individual follow-up. Therefore, the workshops may serve as a complementary intervention tool to facilitate behavioral change.

### Familial Influence on the Development of Eating Habits

The curriculum was developed based on the social-ecological model, considering that individuals’ eating behaviors are influenced by determinants of their environment [71]. Family, as part of their social environment, is one of the most influent determinants of healthy eating in children. Indeed, parents play a crucial role as they usually are responsible for food selection, serve as role models, and use parental feeding practices that impact children’s eating behaviors [72]. According to studies designed for obesity prevention or management in children, family-oriented lifestyle interventions are the most effective in noncancer and in CCS populations [15,73]. Therefore, our curriculum targets patients and their families. It will address the use of positive parental feeding practices [74,75] to promote healthy eating behaviors in children during and after cancer treatments, for example, healthy eating role modeling and avoidance of restrictions or control over eating [9,76].

The curriculum will put forward a positive and total diet approach to healthy eating that considers the whole eating pattern, suggests adding healthy foods instead of forbidding specific foods (apart from those restricted for food safety), and avoids categorization of food as “good” or “bad” [77]. The Mediterranean diet pattern is associated with the prevention of cardiovascular diseases [49] that are frequent long-term complications of CCS [4,5]. Therefore, coupled with Canadian Dietary Guidelines [78], this pattern has guided the development of nutrition education content and recipe criteria.

Food safety is a major concern during pediatric cancer treatment due to weakened immune system. It will be addressed throughout all cooking demonstrations when facilitators will model safe food handling practices. Only little evidence support a neutropenic diet to prevent infection for patients undergoing chemotherapy or radiotherapy, as the only few randomized control trials performed used variable methodologies and presented several limitations [79,80]. Indeed, a neutropenic diet may impose unnecessary food restrictions on patients who often consume insufficient dietary intakes [80,81].

### Considerations Related to Childhood Cancer

Further studies need to evaluate the feasibility of implementing workshops for pediatric oncology patients undergoing cancer treatments. The moment surrounding the diagnosis and treatment of cancer has been described as a *teachable moment* for a healthier lifestyle in adult cancer [82]. However, this opportunity window is not well documented in children. Families overwhelmed with the diagnosis might be less interested or find it too challenging to adopt healthy habits while experiencing a distressing life event [83]. However, focus groups with parents of overweight CCS testing a 6-lesson curriculum after completion of their child’s treatment revealed that some would have preferred to receive the intervention earlier in the process [67], supporting that the timing of our intervention might be optimal.

The heterogeneity of the target population, which comprises children of various ages, diagnoses, and treatments, was a challenge in the development of the curriculum. Evaluating the implementation of the workshops will inform us on participation rate and will allow to calculate sample size of future nutrition education and cooking program in pediatric oncology. The nonrandomized design is also a limitation of this study. Our study was designed to ensure that every newly diagnosed patient could participate and benefit from this novel lifestyle study. Therefore, control participants will only be recruited among patients who completed the standard treatment before the VIE Program was implemented.

We are confident that this intervention will contribute to increase knowledge about nutrition and cooking in the context of childhood cancer. Hopefully, it will improve children’s diet quality while promoting long-term healthy eating habits to prevent cardiometabolic complications. This research- and practice-based nutrition education and cooking demonstration curriculum will be a valuable complement to the VIE Program lifestyle intervention for the prevention of cardiometabolic long-term complications.

## Abbreviations

CCCB: Centre de cancérologie Charles-Bruneau
CCS: childhood cancer survivors
RD: registered dietitian
SJUHC: Sainte-Justine University Health Center
VIE: Valorization, Implication, Education
